# Prediction of Effective Drug Combinations by an Improved Naïve Bayesian Algorithm

**DOI:** 10.3390/ijms19020467

**Published:** 2018-02-05

**Authors:** Li-Yue Bai, Hao Dai, Qin Xu, Muhammad Junaid, Shao-Liang Peng, Xiaolei Zhu, Yi Xiong, Dong-Qing Wei

**Affiliations:** 1 State Key Laboratory of Microbial Metabolism, Joint International Research Laboratory of Metabolic and Developmental Sciences, School of Life Sciences and Biotechnology, Shanghai Jiao Tong University, Shanghai 200240, China; bly1372327795@sjtu.edu.cn (L.-Y.B.); wys8c764@sjtu.edu.cn (H.D.); xuqin523@sjtu.edu.cn (Q.X.); juni_sjtu@sjtu.edu.cn (M.J.); 2 College of Computer Science and Electronic Engineering & National Supercomputing Centre in Changsha, Hunan University, Changsha 410082, China; pengshaoliang1979@163.com; 3 School of Computer Science, National University of Defense Technology, Changsha 410073, China; 4 School of Life Sciences, Anhui University, Hefei 230601, China; xlzhu_mdl@hotmail.com

**Keywords:** drug combination, classification and prediction, improved naïve Bayesian algorithm, metabolic enzyme

## Abstract

Drug combinatorial therapy is a promising strategy for combating complex diseases due to its fewer side effects, lower toxicity and better efficacy. However, it is not feasible to determine all the effective drug combinations in the vast space of possible combinations given the increasing number of approved drugs in the market, since the experimental methods for identification of effective drug combinations are both labor- and time-consuming. In this study, we conducted systematic analysis of various types of features to characterize pairs of drugs. These features included information about the targets of the drugs, the pathway in which the target protein of a drug was involved in, side effects of drugs, metabolic enzymes of the drugs, and drug transporters. The latter two features (metabolic enzymes and drug transporters) were related to the metabolism and transportation properties of drugs, which were not analyzed or used in previous studies. Then, we devised a novel improved naïve Bayesian algorithm to construct classification models to predict effective drug combinations by using the individual types of features mentioned above. Our results indicated that the performance of our proposed method was indeed better than the naïve Bayesian algorithm and other conventional classification algorithms such as support vector machine and K-nearest neighbor.

## 1. Introduction

In the past decades, the “one-drug, one-target” paradigm was dominant in the drug discovery and development phases. Despite large investments, the success rate of the drugs entering into market was significantly lower under the traditional paradigm. The limitation of the present “one-drug, one-target” strategy can be overcome by attacking the disease system using the new systems-oriented drug design strategy (such as “multi-drug, multi-target”), which has become a promising paradigm for the treatment of complex diseases, such as cancer and metabolic disorders [1,2,3]. The therapeutics based on the “multi-drug, multi-target” paradigm can be more efficacious and less vulnerable to adaptive drug resistance by using drug combinations (two or more drugs simultaneously) that impact multiple targets to control complex disease systems [4]. Since the number of possible drug combinations will increase exponentially with a larger number of single drugs reaching the market, it is impractical to determine all effective drug combinations by experimental methods, which are both resource- and time-consuming.

Recently, a number of computational approaches have been proposed for prediction of effective drug combinations [5,6,7,8,9,10,11,12,13,14,15,16,17]. Among these approaches, a wide variety of drug-related information was utilized to identify potential feature patterns enriched in approved drug combinations. The information to represent or describe drugs and the pairs of drugs can include the features derived from drug chemical structure-based fingerprints and drug similarity, the target proteins of drugs, drug indications, pathways possibly affected by a drug through its targets, therapies encoded by the Anatomical Therapeutic Chemical (ATC) code, and side effects reported in clinical findings. The hypothesis or conclusions based on the features mentioned above, which were supported by the data set of known drug combinations, can provide crucial clues to the latent molecular mechanisms of effective or beneficial drug combinations. For example, Bork et al. observed that protein pairs targeted by newly approved drug combinations were frequently or repeatedly used as the targets of previously approved drug combinations [6]. Zhao et al. indicated that effective drug combinations were more likely to modulate functionally related pathways and inclined to have a smaller effect radius in the genetic interaction networks than that of the random combinations [5]. Lu and Cao et al. came to the conclusion that drug pairs acting on the same pathway through different targets or drugs regulating a relatively small number of highly-connected pathways were more likely to be synergistic drug combinations [12]. Cai et al. discovered that the chemical-chemical interaction between drug pairs and protein-protein interactions between their targets were crucial for determining whether a drug pair is an effective combination [7]. Based on the potentially useful features, the simple scoring function or sophisticated machine learning algorithms, such as random forest [7], support vector machine [8], stochastic gradient boosting algorithm [9], and Bayesian network [13], were used to build the prediction models by integrating various features to distinguish the effective drug combinations from random drug pairs. Moreover, in the last decade, there have been a number of published studies and reviews on the application of quantitative structure-activity relationship (QSAR)-based methods in the fields of chemometrics and chemoinformatics [18,19,20,21,22,23,24].

Although the existing methods can predict the effective drug combinations, all of them concentrated only on pharmacological, molecular or phenotypic information of drug pairs. It is well known that drugs will go through four steps (i.e., absorption, distribution, metabolism and excretion), when they enter the human body [25]. In this study, we not only used the common features in previous studies, but also took information about the enzymes and transporters of drugs into consideration. Therefore, we used target, pathway, side effects, enzyme and drug transporter information to analyze and predict effective drug combinations. To reduce the redundancy among features and extract informative features, a commonly used feature selection method of Minimum Redundancy Maximum Relevance (mRMR) [26] and a new feature selection method were utilized to decrease the computational time and complexity of the prediction model. Then, we devised an improved Naïve Bayesian algorithm to build the classification model, which was compared with the models by using the conventional algorithms such as support vector machine (SVM), naïve Bayesian (NB), K-nearest neighbor (KNN). Our results indicated that the improved naïve Bayesian algorithm yielded better performance than other algorithms in this prediction task. In addition, it was found that the information about enzymes of drugs was definitely important for predicting effective drug combinations.

## 2. Results and Discussion

### 2.1. Coverage of Drug Combinations by Different Features

**Figure 1:**
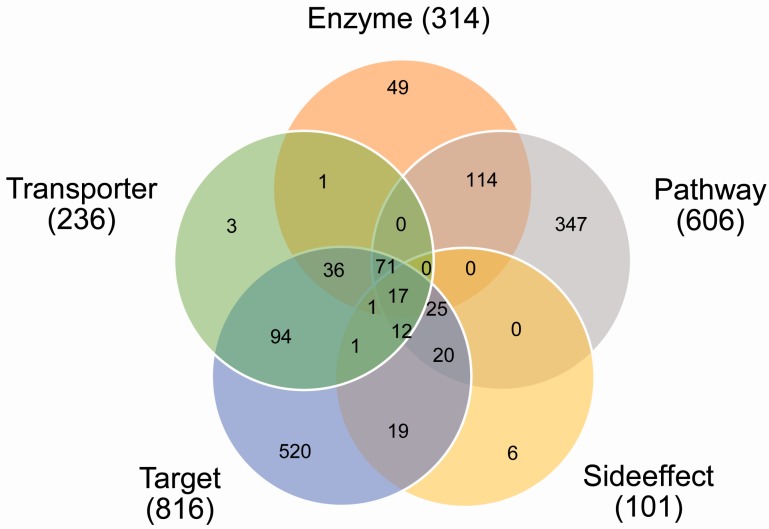
The Venn diagram of drug combinations for five types of features, where the numbers show how many drug pairs (there were a total of 946 drug pairs) can be covered by different features or combinations of these feature types.

Because of the incomplete coverage of drug pairs by different sources of features, some types of feature values were not available for the given drug pairs. For the five single types of features and any possible combination of these feature types, we counted the number of drug pairs which had the available values of the corresponding feature types. As shown in Figure 1, most of the drug pairs had the available information about the targets and pathways in which their targets were involved, indicating that the targets and pathways of drugs were extensively studied, in comparison to the other types of information of the drugs.

### 2.2. The Impact of Different Ratios of Positive-to-Negative Samples on the Classification 

The negative samples (non-effective drug combinations) were generated by randomly pairing drugs that appeared in the data set of positive samples (effective drug combinations). In this section, we randomly sampled different numbers of non-effective drug combinations to build the classification models to check whether the ratios of positive-to-negative samples can affect the prediction performance. The three positive-to-negative sample ratios were 1:1, 1:2, and 1:3. We used 75% of all positive and negative samples to train the classification model, and made the prediction on the remaining 25% of the samples as the independent test. As shown in Table 1, the overall accuracy remained similar and the other measures decreased as the number of negative samples increased. In the remaining sections, we used the same number of negative samples as the positive ones in the training and testing sets.

### 2.3. The Impact of Different Compositions of Negative Samples on the Classification

It is a non-trivial task to construct a benchmark negative data set, since non-effective drug combinations were seldom reported in the literature, and few of these pairs were reported in a publicly known database. The aim of this section is to test whether the different compositions of negative data sets have an impact on the classification performance. We constructed the predictive models based on two different data sets. Both data sets had the same compositions of positive samples of the effective drug combinations, which had been approved and used in clinical treatments. However, they had different compositions of negative samples, which were collected from two different sources. The first one (N1) was based on the random combination of two single drugs which appeared in the positive pairs, whereas the second one (N2) was based on the drugs from a drug combination database (DCDB) [27]. In both negative sets, the combinations that appeared in positive samples would not appear in negative samples.

**Figure 2:**
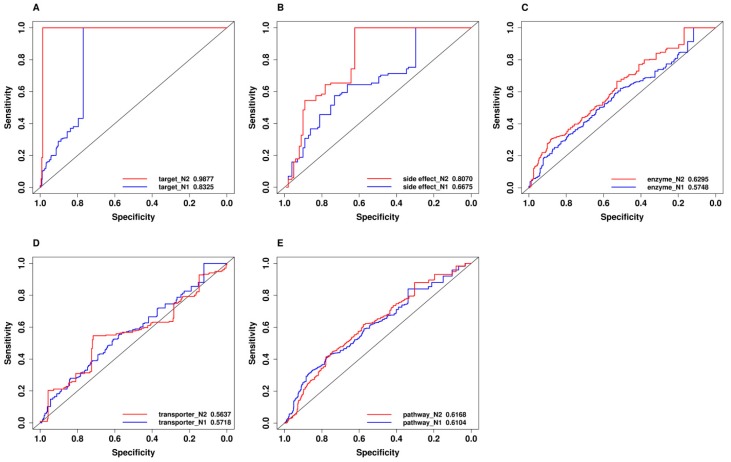
The performance comparison of models on two different negative data sets by using leave-one-out cross validation on five types of single features. The performance of the N1 negative data set is shown by the blue line, whereas the performance of the N2 negative data set is shown by the red line: (**A**) The enzyme feature; (**B**) The pathway feature; (**C**) The side effect feature; (**D**) The transporter feature; (**E**) The target feature.

The two predictive models that were based on two different negative data sets yielded different classification performance (Figure 2). The detailed evaluation measures can be found in Table S1 (on N1 negative data set) and Table S2 (on N2 negative data set). The model that was built based on the second negative data set achieved better performance as compared to the model that was built based on the first negative set, regardless of the input features such as drug target, the side effect and metabolic enzymes. These results can be explained by the fact that single drugs in the positive pairs were frequently used in the proven effective drug combinations. The first negative data set may have the true positive ones to be experimentally determined, which leads to its weak classification performance, whereas the second negative data set was randomly generated by all the single drugs in the DCDB database. The possibility for the N2 data set with the probable effective drug combinations was smaller than that of the N1 data set. The fact that a drug which appeared in proven effective drug combinations is more likely to be combined with other drugs to become effective drug combinations, is consistent with the findings in the previous study [6].

### 2.4. Comparison of Different Feature Selection and Classification Algorithms

**Figure 3:**
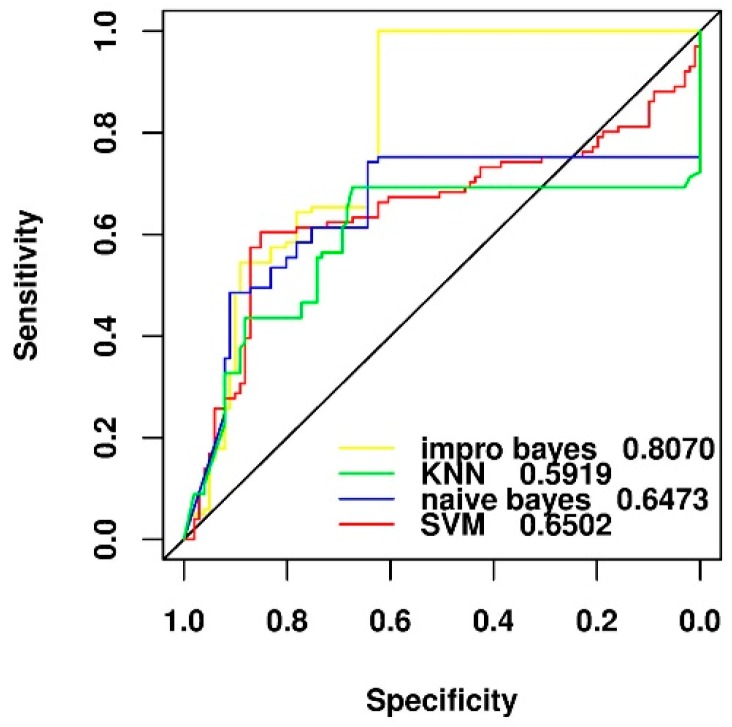
Performance comparison of different machine learning algorithms for the N2 negative data set by using our feature selection method of leave-one-out cross validation test.

In this section, we aimed to compare the classification performance of four different machine learning algorithms (KNN, SVM, naïve Bayesian, and improved naïve Bayesian) using the feature of side effect of drugs. As shown in Figure 3, the improved naïve Bayesian algorithm with the area under the curve (AUC) of receiver operating characteristic (ROC) as 0.8070 performed better than the three other types of traditional algorithms, which achieved similar performance, with the AUC values of ROC ranging from 0.5919 to 0.6502.

### 2.5. Comparison of Two Different Feature Selection Methods

**Figure 4:**
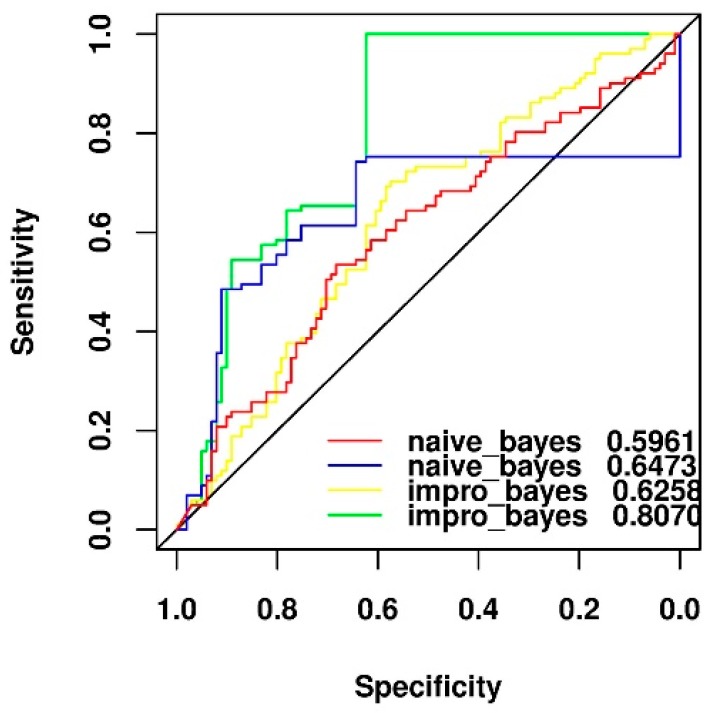
Performance comparison of two different feature selection algorithms for the N2 negative data set by using the naïve Bayesian and improved naïve Bayesian method of the leave-one-out cross validation test. The blue and green lines used our new method to select features, while the red and yellow lines used the mRMR algorithm for feature selection.

We used two different feature selection methods with two types of Bayesian algorithms to build four different models using the feature of side effect of drugs on the N2 negative data set. It was observed from Figure 4 that regardless of the type of Bayesian algorithm, the result of our proposed feature selection method (AUC as 0.6473 and 0.8070) was better than that of the mRMR approach (AUC as 0.5961 and 0.6258) with respect to the classification task. Moreover, regardless of the feature selection method, the result of the improved naïve Bayesian (AUC as 0.6258 and 0.8070) was better than that of the naïve Bayesian algorithm (AUC as 0.5961 and 0.6473). Therefore, the new feature selection method embedded in the improved naïve Bayesian algorithm was finally used to build the prediction model in this work.

### 2.6. Performance Comparison of Original and y-Randomization Data Sets

**Figure 5:**
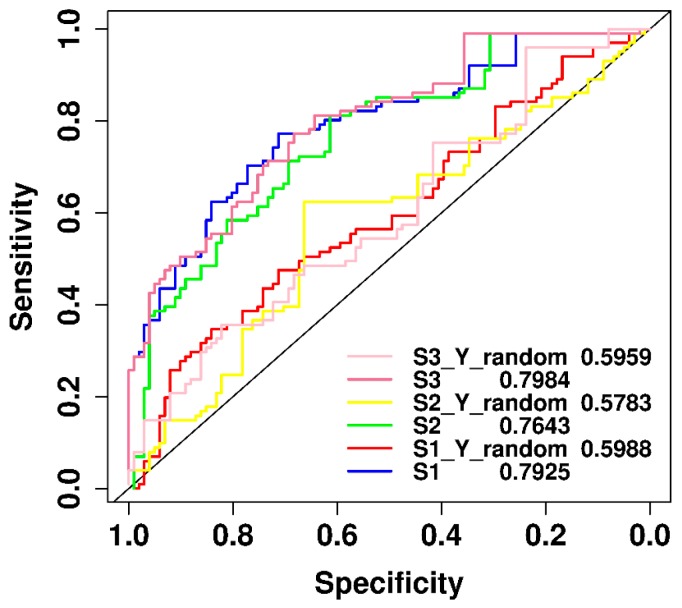
Performance comparison between the original models (S1, S2, S3) and the models with randomly shuffled data sets (S1_Y_random, S2_Y_random, S3_Y_random).

The aim of this section is to compare the classification performance of the original model to that of the models built with permuted (randomly shuffled) samples. We used the improved naïve Bayesian algorithm with the feature of side effects for the N2 negative data set and its variants to construct and evaluate the models by the leave-one-out cross validation test. As shown in Figure 5, the classification performances of our original models were much better than those of the models built for randomly shuffled data sets. The results demonstrated that our models developed in this work obtained good predictive power and stability, much better than by pure chance.

### 2.7. Comparison of Predictive Power of Individual Features

**Figure 6:**
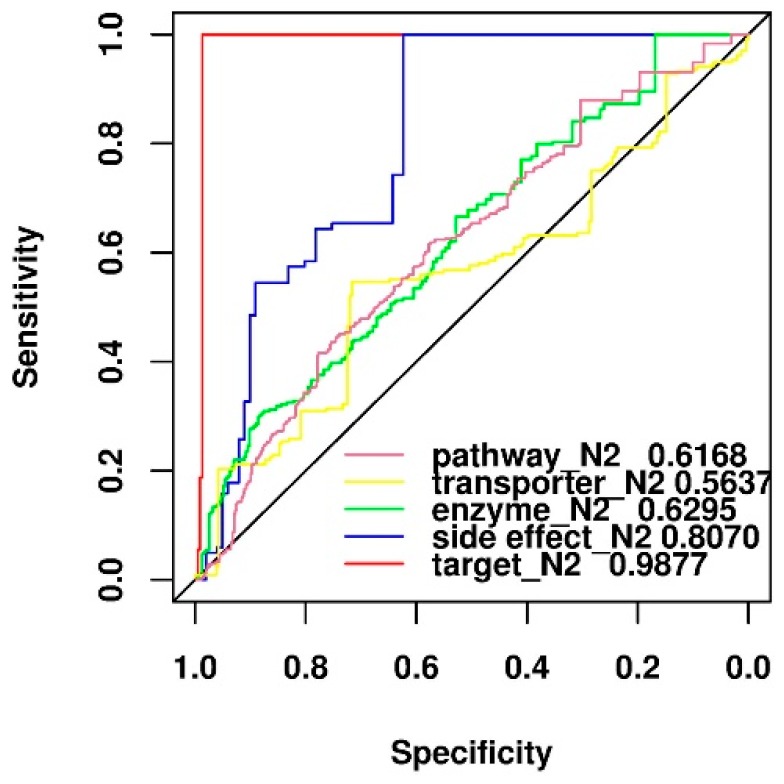
Performance comparison of five different feature types for the N2 negative data set by using our feature selection and improved naïve Bayesian method in the leave-one-out cross validation test.

In this section, we aimed to assess and compare the prediction performances of the five types of features using our feature selection and improved naïve Bayesian algorithms for the N2 negative data set. It is clear from Figure 6 that the feature based on drug targets had the highest predictive performance among the five types of features. This result can be explained by the fact that target protein pairs are frequently used in the curated data set of drug combinations, which is in agreement with the findings of a previous study [6]. The clinical side effect feature demonstrated good performance of the prediction of effective drug combinations, which achieved a similar level of performance as a previous study [16]. Among the two newly proposed features (enzyme- and transporter-based features), the enzyme-based feature performed better than the conventional feature of pathway information. A similar tendency was achieved on the independent data set on the five types of features (shown in Table 2).

### 2.8. Comparison with Previously Published Methods

**Figure 7:**
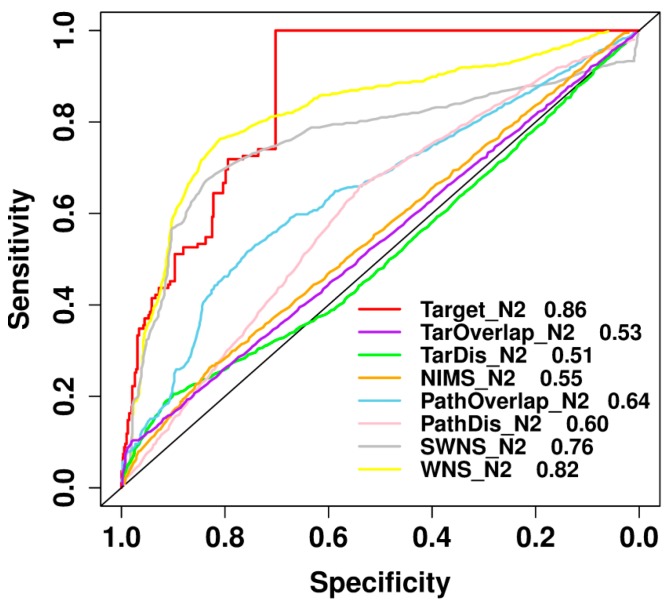
Performance comparison between our proposed method and Chen et al.’s method by using the leave-one-out cross validation test [12]. The red line shows the classification performance of our method while the others show the performances of Chen et al.’s models.

Using the same data set used in Chen et al.’s study [12], we compared our method based on the feature of target information to the models in Chen et al.’s study, which used a synergy evaluation method by a pathway-pathway interaction network to predict the potential synergistic drug combinations. The performance comparison is shown in Figure 7. The results demonstrated that our method performed better than that of Chen et al.’s models. This improvement can be mainly attributed to two reasons. Firstly, our method directly used the feature based on the targets of drugs, which were greatly helpful to predict effective drug combinations, whereas Chen et al.’s method used the pathway-pathway interactions from target proteins of drugs. Secondly, our method used a sophisticated machine learning method to predict drug combinations, while Chen et al. used a simple method by setting a threshold to determine whether a pair of drugs was a synergistic drug combination.

## 3. Materials and Methods

### 3.1. Data Sets

#### 3.1.1. Positive Data Set

The drug combination information was retrieved from the DCDB database (http://www.cls.zju.edu.cn/dcdb/), in which the entries were collected from the food and drug administration (FDA) electronic orange book and published clinical studies. A total of 1363 records of successful drug combinations (a drug combination is a recipe of two or more drugs) was retrieved. In this study, we focused on 946 drug combinations, each of which consists of two single drugs (drug pairs) simultaneously. The information regarding enzymes, transporters, targets, pathways and side effects of drugs was extracted from different sources. The information for the enzymes, transporters and targets of drugs was obtained from the DrugBank database (https://www.drugbank.ca/) [28]. The pathway-based information that is affected by the drugs through target modulation was extracted from the KEGG database (http://www.kegg.jp/kegg/pathway.html) [29]. The side effect information was retrieved from the SIDER database (http://sideeffects.embl.de/) [30]. Only those drug pairs that had the available feature information for each of its drug components were subjected to further analysis. Finally, the drug pairs were collected and grouped as below. They included: (1) Group 1, denoted as “G1”, which contained 101 drug combinations (including 120 single drugs) that had the available side effect information; (2) Group 2, denoted as “G2”, which contained 314 drug combinations (including 262 single drugs) that had the available enzyme information; (3) Group 3, denoted as “G3”, which contained 236 drug combinations (including 201 single drugs) that had the available transporter information; (4) Group 4, denoted as “G4”, which contained 816 drug combinations (including 628 single drugs) that had the available target information; (5) Group 5, denoted as “G5”, which contained 606 drug combinations (including 486 single drugs) that had the available pathway information.

#### 3.1.2. Negative Data Sets

As inspired by Chen et al.’s work [12], we constructed two different types of negative data sets. The first data set, denoted as “N1”, was generated by randomly sampling any two of the single drugs, which were involved in the positive data set of drug combinations, for each group defined in the previous section. The second data set, denoted as “N2”, was generated from the random pairs of all possible single drugs from the whole DCDB database. Then, we collected enzyme, side effect, pathway, target and transporter information of these drug pairs in the negative data set, in a similar way as that of the positive data set.

### 3.2. Feature Sets

#### 3.2.1. Feature Representation

In this study, three major categories of features were used to represent drug pairs, which included pharmacodynamics, pharmacokinetic and phenotypic properties. For the absence or presence of any target, pathway, metabolic enzyme, drug transporter, or side effect, the corresponding element of a drug vector was encoded as either 1 or 0 [31]. Therefore, for a pair of drugs, the element was encoded as the sum of the elements of two single drugs, which can be 2, 1 or 0. However, for the feature of side effect, the SIDER database collected the side effect data under high and low doses of the given drug. Therefore, the side effect element of a drug combination was probably 4, 3, 2, 1 or 0 (high dose of the drug was 2, low dose of the drug was 1, no side effect was 0). Table 3 shows the total bits of each type of feature vector for the two different negative data sets.

#### 3.2.2. Feature Selection

As suggested by Zou et al. [32,33], the effective dimensionality reduction of features can decrease the computational time and complexity of the prediction model and also provide more insight into the data abundance. In the present work, we implemented the feature selection using the Minimum Redundancy Maximum Relevance (mRMR) method [26] and a new method of feature selection in the improved naïve Bayesian. For each model, we finally chose 30 features from drug targets, pathways, side effect, metabolic enzymes, and drug transporters.

The new method of feature selection embedded in the improved naïve Bayesian algorithm is described as below:

(1) Basic assumptions: In the selection of features, for the convenience of calculation, we set all the features to obey a 0–1 distribution. If a value of the feature element was not 0, no matter how much it was, we took the value as 1.
(2) Basic principles: In the negative samples, we set the frequency $p_{1}$ for the feature element value to 1; while in the mixed positive and negative samples, we set the frequency $p_{2}$ for the feature element value to 1. If there was no difference in the positive and negative samples, the frequency $p_{2}$ and $p_{1}$ should be approximately equal; while if the difference between $p_{1}$ and $p_{2}$ was beyond a certain level of significance, the feature was considered as the important one.
(3) Methods: For all the features, $p_{1}$ was the abscissa, $p_{2}-p_{1}$ was the ordinate, and a scatter diagram was drawn.

If there was no difference between the distribution of positive and negative samples, it was found that under the same level of significance, the scatter should fall within the following ellipse based on a large number of numerical simulations:

(1) $$\frac{{\left(p_{1}-0.5\right)}^{2}}{0.5^{2}}+\frac{{\left(p_{2}-p_{1}\right)}^{2}}{a^{2}}=1$$

The different values of *a* represent the different significance levels. If there is a significant difference between the distribution of positive and negative samples, scattered points will fall outside the ellipse.

### 3.3. Model Construction

#### 3.3.1. Classification Algorithms

In this study, we adopted the prediction of effective drug combinations as a binary classification task. For each type of feature in the corresponding data set, we built classification models using various machine learning algorithms, which included support vector machine, K-nearest neighbor, naïve Bayesian, and improved naïve Bayesian. In the SVM algorithm, we employed Gaussian kernel as the kernel function, and the cost factor *C* and gamma *γ* were optimized by a grid search (i.e., the optimal parameters at *C* = 10, and *γ* = 0.1). In the KNN algorithm, the parameters of *k* (the number of neighbors considered) and *l* (the minimum vote for definite decision), were optimized by a grid search (i.e., the optimal parameters at *k* = 5, and *l* = 2). In the NB algorithm, the parameter of Laplace smoothing was set to zero. The leave-one-out cross validation test was employed to build different classification models for comparison.

In order to test the models independently, we combined the positive data set and negative data set as a whole data set, and then randomly split the whole data set into two parts (i.e., 75% of the data set for training and 25% for testing). We also sampled different sizes of negative data sets to test what size of the negative data set yielded better performance.

#### 3.3.2. Improved Naïve Bayesian Algorithm

We proposed an improved Bayesian method, which used the distribution of the sample properties and their relevance as prior knowledge to join in the process of model construction. This method avoided the assumption in the naïve Bayesian method that the conditional probability is independent. It not only took advantage of the biological activity of the samples but also made full use of a larger group of common data whose biological activities are unknown. For example, if we had 1000 candidates of drug combinations, the corresponding enzyme information of only 100 candidates was known and the activities of the other 900 candidates were unknown. In most methods, they only used 100 drug combinations, whereas in our improved Bayesian method, we also used the other 900 candidates. According to this set of common data, we can estimate the distribution type of the sample properties and the correlation between various properties, but these factors generally had nothing to do with the biological activity of samples. By adding it as prior knowledge into the process of model construction, it is used to consider the correlation between attributes in advance.

This method is suitable for the biological data set which had a limited sample size but a large number of sample properties. The method can reduce the instability and the over-fitting problem brought about by the small sample data and yield a better prediction effect for this kind of data.

The outline of the proposed algorithm is listed below:

(1) We applied normal transformation to the features of all samples, $X_{1},X_{2},\cdots ,X_{n}$ and then obtained a new set of attributes $\hat{X_{1}},\hat{X_{2}},\cdots ,\hat{X_{n}}$; all these distributions obey the normal distribution.
(2) According to the new set of attribute values, we calculated the correlation coefficient matrix R and calculated the characteristic values of $\lambda_{1},\lambda_{2},\cdots ,\lambda_{n}$ and corresponding eigenvector of $\alpha_{1},\alpha_{2},\cdots ,\alpha_{n}$.
(3) We calculated the mean value $\mu_{k1},\mu_{k2},\cdots ,\mu_{kn}$ and the standard deviation $\sigma_{k1},\sigma_{k2},\cdots ,\sigma_{kn}$ of transformed attributes in category $C_{k}$ and calculated the mean value $\mu_{k1},\mu_{k2},\cdots ,\mu_{kn}$ and the standard deviation $\sigma_{k1},\sigma_{k2},\cdots ,\sigma_{kn}$ of transformed attributes for all samples. Let $P=\left(\alpha_{1},\alpha_{2},\cdots ,\alpha_{n}\right);$ then, for each sample $\hat{x}=\left(\hat{x_{1}},\hat{x_{2}},\cdots ,\hat{x_{n}}\right)$, calculated as:
  (2) $$\left(\beta_{1},\beta_{2},\cdots ,\beta_{n}\right)=\left(\begin{array}{cccc} \frac{\hat{x_{1}}-\mu_{1}}{\sigma_{1}}, & \frac{\hat{x_{2}}-\mu_{2}}{\sigma_{2}}, & \cdots , & \frac{\hat{x_{n}}-\mu_{n}}{\sigma_{n}} \end{array}\right)P$$
  (3) $$\left(\gamma_{1},\gamma_{2},\cdots ,\gamma_{n}\right)=\left(\begin{array}{cccc} \frac{\hat{x_{1}}-\mu_{k1}}{\sigma_{k1}}, & \frac{\hat{x_{2}}-\mu_{k2}}{\sigma_{k2}}, & \cdots , & \frac{\hat{x_{n}}-\mu_{kn}}{\sigma_{kn}} \end{array}\right)P$$
(4) If the prior probability $P\left(C_{k}\right)$ is very clear, for each category $C_{k}$:
  (4) $$H_{k}=\ln P\left(C_{k}\right)-\overset{n}{\underset{i=1}{\sum }}\left(\frac{{\gamma_{i}}^{2}}{2\lambda_{i}}+\ln\sigma_{ki}\right)$$
  $\mathrm{when}\;H_{k}$ is the maximum value, the corresponding category $C_{k}$ is the predicted category of the sample.
(5) If the prior probability $P\left(C_{k}\right)$ is not clear, then for a certain category $C_{k}$, calculated on all samples:
  (5) $$L=\overset{n}{\underset{i=1}{\sum }}\left(\frac{{\beta_{i}}^{2}-{\gamma_{i}}^{2}}{\lambda_{i}}\right)$$

The greater the value of *L*, the greater the probability the sample belongs to category $C_{k}$. According to the *L* values of all samples and whether a given sample belongs to $C_{k}$, a receiver operating characteristic curve was drawn, and then the appropriate threshold was chosen and it sas determined whether each sample belonged to category $C_{k}$.

### 3.4. y-Randomization Test

The *y*-Randomization test is often used for validation of QSAR models [34]. In this study, we compared the performance of the original models to that of the classification models with randomly shuffled data sets by *y*-Randomization. We sampled the negative data set three times from the N2 negative data set, whose size was the same as that of the positive data set. Then, we randomly shuffled the labels of all samples in the whole data set (positive and negative data set together), and constructed the classification models based on the newly shuffled data sets.

### 3.5. Model Evaluation

The classification performances of prediction models were evaluated by the metrics such as accuracy, recall (also called sensitivity), specificity, precision, F-measure, and Matthew’s correlation coefficient (MCC), which are defined as below:

(6) $$\mathrm{Accuracy}=\frac{TP+TN}{TP+FP+TN+FN}$$

(7) $$\mathrm{Recall}=\frac{TP}{TP+FN}$$

(8) $$\mathrm{Specificity}=\frac{TN}{TN+FP}$$

(9) $$\mathrm{Precision}=\frac{TP}{TP+FP}$$

(10) $$F-\mathrm{measure}=\frac{2\times Recall\times Precision}{Recall+Precision}$$

(11) $$\mathrm{MCC}=\frac{TP\times TN-FP\times FN}{\sqrt{\left(TP+FP\right)\times \left(TN+FN\right)\times \left(TP+FN\right)\times \left(TN+FP\right)}}$$

where TP is the number of correctly predicted effective drug combinations, TN is the number of correctly predicted non-effective drug combinations, FP is the number of non-effective drug combinations predicted as effective ones, and FN is the number of effective drug combinations wrongly predicted as non-effective ones [35,36]. These metrics have also been used by a series of studies [37,38,39,40,41,42,43,44,45,46,47,48,49]. Moreover, a receiver operating characteristic curve was plotted by the sensitivity versus (1-specificity) for a binary classifier at dynamic thresholds ranging from 0 to 1. The area under the curve was used as an overall measure to evaluate the predictive performances of the classification models.

## 4. Conclusions

The “multi-drug, multi-target” paradigm has attained increasing popularity in the treatment of complex diseases, such as cancer and metabolic disorders, since it can be more efficacious and less vulnerable to the adaptive drug resistance by using drug combinations (two or more drugs simultaneously) that impact multiple targets to control complex disease systems. For simplicity, we focused on the drug combinations that consisted of only two single drugs in this work. Since experimental determination of all possible drug combinations is both resource- and time-consuming, there is an urgent need to develop computational methods for prediction of drug combinations.

In the present study, we firstly conducted systematic analysis of various types of features for representation of drug combinations. These features included the traditional features such as targets of drugs, the pathway in which the drug’s target was involved, and the side effect of drugs, and the newly proposed features, such as metabolic enzymes of drugs, and transporters of drugs, which are related to the metabolism of drugs. Our results indicated that the feature based on drug targets yielded the best performance, reflecting that fact that the target protein pairs are frequently used in the curated data set of drug combinations. The clinical side effect feature performed well, based on the assumption that the drug pairs that can be co-administrated often do not have the same or similar adverse drug reactions. The novel feature of the enzyme-based information demonstrated better performance than that of the conventional feature such as pathway information, indicating the important role of metabolic enzymes of drugs in prediction of the drug combinations.

Then, we proposed an improved naïve Bayesian algorithm for prediction of effective drug combinations. The new method is suitable for small data sets and is able to construct more stable and accurate predictive models compared with other machine learning models. Our experimental results indicated that the improved naïve Bayesian algorithm achieved better performance than the other machine learning algorithms such as SVM, KNN, and naïve Bayesian algorithms in this prediction task. We believe that our proposed method will be potentially useful in large-scale in silico drug combination screening. However, it should be pointed out that the present study does not cover every aspect of drug combinations, and the research is ongoing and will provide further improvements such as an attempt to integrate various features together in future studies.

## Figures and Tables

**Table 1:** The classification performance of different positive-to-negative sample ratios by using the feature of the side effect of drugs on the independent test.

Positive-to-Negative Ratio	Accuracy	F-Measure	MCC	Recall	Precision
1:1	0.6800	0.6667	0.3612	0.6400	0.6957
1:2	0.6667	0.5098	0.2638	0.5652	0.4643
1:3	0.6832	0.3043	0.0992	0.3043	0.3043

**Table 2:** Performance comparison of prediction models based on different feature types using the improved naïve Bayesian algorithm on the independent data set.

Feature Type	Accuracy	F-Measure	MCC	Recall	Precision
Targets	0.7034	0.6431	0.4771	0.5000	0.9008
Side effect	0.6800	0.6667	0.3612	0.6400	0.6957
Pathways	0.6238	0.6174	0.2474	0.6216	0.6133
Enzymes	0.6115	0.6904	0.2144	0.8095	0.6018
Transporters	0.5339	0.5865	0.1216	0.7500	0.4815

**Table 3:** The dimensions of different features for the two different negative data sets.

Category	Feature	Source	Dimension (N1)	Dimension (N2)
Pharmacodynamics	Targets	DrugBank	681	787
	Pathways	KEGG	255	263
Pharmacokinetic	Enzymes	DrugBank	135	146
	Transporters	DrugBank	76	86
Phenotypic	Side effect	SIDER	3005	3889

## Supplementary Materials

The following are available online at http://www.mdpi.com/1422-0067/19/2/467/s1.
