# Supplementary material for: Prediction of Effective Drug Combinations by an Improved Naïve Bayesian Algorithm
Source: Int J Mol Sci. 2018 Feb 5;19(2):467. doi: 10.3390/ijms19020467 (PMC5855689; doi:10.3390/ijms19020467)
Supplement: Supplementary file 1 [file ijms-19-00467-s001.pdf]

Supplementary

Prediction of Effective Drug Combinations by an Improved Naïve Bayesian Algorithm

Li-Yue Bai [1], Hao Dai [1], Qin Xu [1], Muhammad Junaid [1], Shao-Liang Peng [2,3], Xiaolei Zhu [4], Yi Xiong [1,*] and Dong-Qing Wei [1,*]

[1] State Key Laboratory of Microbial Metabolism, Joint International Research Laboratory of Metabolic and Developmental Sciences, School of Life Sciences and Biotechnology, Shanghai Jiao Tong University, Shanghai 200240, China; bly1372327795@sjtu.edu.cn (L.-Y.B.); wys8c764@sjtu.edu.cn (H.D.); xuqin523@sjtu.edu.cn (Q.X.); juni_sjtu@sjtu.edu.cn (M.J.)

[2] College of Computer Science and Electronic Engineering & National Supercomputing Centre in Changsha, Hunan University, Changsha 410082, China; pengshaoliang1979@163.com

[3] School of Computer Science, National University of Defense Technology, Changsha 410073, China

[4] School of Life Sciences, Anhui University, Hefei 230601, China; xlzhu_mdl@hotmail.com

* Correspondence: xiongyi@sjtu.edu.cn (Y.X.); dqwei@sjtu.edu.cn (D.-Q.W.); Tel.: +86-21-3420-4573 (Y.X. & D.-Q.W.)

Table S1. Performance comparison of prediction models based on different feature types by using leave-one-out cross validation test on N1 negative data set.

Feature type	Accuracy	F-measure	MCC	Recall	Precision	AUC
Target	0.8848	0.8967	0.7909	1	0.8127	0.8325
Side effect	0.6485	0.7399	0.4176	1	0.5872	0.6675
Enzyme	0.5621	0.5802	0.1247	0.6051	0.5572	0.5748
Pathway	0.5883	0.4336	0.2108	0.3152	0.6945	0.6104
Transporter	0.553	0.4604	0.1128	0.3814	0.5806	0.5718

Table S2. Performance comparison of prediction models based on different feature types by using leave-one-out cross validation test on N2 negative data set.

Feature type	Accuracy	F-measure	MCC	Recall	Precision	AUC
Target	0.9933	0.9933	0.9866	1	0.9867	0.9877
Side effect	0.8119	0.8417	0.6732	1	0.7266	0.807
Enzyme	0.5908	0.6532	0.1946	0.7706	0.5667	0.6295
Pathway	0.5949	0.5035	0.2041	0.4109	0.6501	0.6168
Transporter	0.5763	0.5868	0.1527	0.6017	0.5726	0.5637
